# A test of the predictive validity of relative versus absolute income for self-reported health and well-being in the United States

**DOI:** 10.4054/demres.2023.48.26

**Published:** 2023-05-16

**Authors:** David Brady, Michaela Curran, Richard M. Carpiano

**Affiliations:** 1 University of California, Riverside, USA, and WZB Berlin Social Science Center, Germany.; 2 Centers for Disease Control, USA.; 3 University of California, Riverside, USA.

## Abstract

**BACKGROUND:**

A classic debate concerns whether absolute or relative income is more salient. *Absolute* values resources as constant across time and place while *relative* contextualizes one’s hierarchical location in the distribution of a time and place.

**OBJECTIVE:**

This study investigates specifically whether absolute income or relative income matters more for health and well-being.

**METHODS:**

We exploit within-person, within-age, and within-time variation with higher-quality income measures and multiple health and well-being outcomes in the United States. Using the Panel Study of Income Dynamics and the Cross-National Equivalent File, we estimate three-way fixed effects models of self-rated health, poor health, psychological distress, and life satisfaction.

**RESULTS:**

For all four outcomes, relative income has much larger standardized coefficients than absolute income. Robustly, the confidence intervals for relative income do not overlap with zero. By contrast, absolute income mostly has confidence intervals that overlap with zero, and its coefficient is occasionally signed in the wrong direction. A variety of robustness checks support these results.

**CONCLUSIONS:**

Relative income has far greater predictive validity than absolute income for self-reported health and well-being.

**CONTRIBUTION:**

Compared to earlier studies, this study provides a more rigorous comparison and test of the predictive validity of absolute and relative income that is uniquely conducted with data on the United States. This informs debates on income measurement, the sources of health and well-being, and inequalities generally. Plausibly, these results can guide any analysis that includes income in models.

## 1. Introduction

Decades of research have established that economic resources contribute to health and well-being (Brady, Kohler, and Zheng 2023; Chin 2010; Deaton 2014; D’Ambrosio, Jantti, and Lepinteur 2020; Franks and Gold 2003; Headey, Muffels, and Wooden 2008; Jebb et al. 2018; Kahneman and Deaton 2010; Link and Phelan 1995; Mackenbach et al. 1997; Manor, Matthews, and Power 2000). Income specifically has been linked with a variety of health outcomes across diverse populations and settings (Ladin, Daniels, and Kawachi 2010; Marmot and Wilkinson 2001; Mackenbach et al. 2005; Subramanyam et al. 2009). Income buys access to health care; purchases food, housing, and other necessities; cultivates security; and raises one’s status and connections with others. Regardless of whether the relationship is causal and/or reciprocal, considerable evidence shows that income predicts health and well-being. Despite this consensus, a classic debate between absolute and relative income remains unresolved (Asadullah and Chaudhury 2012; Ball and Chernova 2007; Corazzini, Esposito, and Majorano 2012; Dohmen et al. 2011; Link, Carpiano, and Weden 2013).

Through two key innovations, this study provides a unique test to adjudicate this debate. First we exploit within-person, within-age, and within-time variation within the United States specifically. Second we use higher-quality, state-of-the-art income measures, incorporating taxes and transfers and adjusting for household size. Incorporating these advances with multiple health and well-being outcomes, we provide a more rigorous comparison and test of the predictive validity of absolute and relative income that is uniquely conducted with data on the United States.

## 2. Absolute versus relative income

The debate between absolute and relative income is critical for understanding inequalities and for normative debates. The debate also speaks to absolute and relative poverty measures, income interventions designed to improve health and well-being, and literatures on social class. As Sen (1999) argues, economic resources are different than well-being. We care about economic resources more because of their extrinsic rather than intrinsic utility. Resources matter because they enable people to pursue and purchase well-being. Hence a better measure of economic resources should better predict health and well-being. As a result, this debate is also relevant to the huge quantity of studies including income in their analyses. For scholars including income in their models, it would be useful to know how best to operationalize income. To the extent that either absolute income or relative income is more important to health and well-being, this provides concrete empirical evidence on which indicator has greater predictive validity.

### Absolute income

Absolute income measures resources independent of time and place. If a person or place gets richer and living standards rise, health and well-being should mechanically improve as absolute incomes increase (Chin 2010; Stevenson and Wolfers 2013). This implies that population health depends most on rising economic development and mean incomes and that those improvements alleviate material deprivation and meet basic needs (Deaton 2014). If absolute income is more important than relative income, than health and well-being depend on one’s fixed material resources almost regardless of others’ material resources in a community or society (Diener et al. 1993; Dolan, Peasgood, and White 2008; Ladin, Daniels, and Kawachi 2010; Veenhoven 1991).

### Relative income

Relative income locates one relationally within a distribution of a time and place. Income is a positional good, and its value depends on one’s hierarchical rank compared to others in a given cultural and historical context (Jebb et al. 2018). Income is a basis for esteem, status, and prestige compared to others in one’s setting (Alderson and Katz-Gerro 2016; Hastings 2019). Moreover, the living standards in one’s cultural and historical context determine what gets defined as a “need” (Smeeding 2016). Hence having sufficient resources to meet one’s needs is always relative (Fox, Torche, and Waldfogel 2016; Gustafsson and Sai 2020; Rainwater and Smeeding 2004). If a country’s living standards and mean incomes rise, health and well-being will not necessarily improve if one’s standing among others declines or stagnates (Avendano 2012; Easterlin et al. 2010). Also, inequality and relative deprivation could undermine the relationship between economic development and population health (Curran and Mahutga 2018; Hastings 2019; Ladin, Daniels, and Kawachi 2010; Rambotti 2015). If relative income is more important than absolute income, health and well-being depend most on one’s hierarchical position compared to others within a context (Alderson and Katz-Gerro 2016; Boyce, Brown, and Moore 2010; Brady et al. 2022; Brown, Gathergood, and Weber 2017; Christoph 2010; Hounkpatin et al. 2015; Marmot and Wilkinson 2001; Townsend 1980).

## 3. Past research

Four kinds of studies predominate in this debate. First, laboratory-style experiments simulate absolute and relative resources and/or consumption (Alpizar, Carlsson, and Johansson-Stenman 2005; Diener et al. 1993; Dohmen et al. 2011; Jachimowicz et al. 2020). Second, observational studies evaluate the health impact of subjective perceptions of relative deprivation (Mishra and Carleton 2015; Stewart 2006). Third, an extensive literature uses ecological analyses testing measures of inequality or relative poverty against measures of economic development or absolute poverty across countries, states, and communities (Beckfield 2004; Curran and Mahutga 2018; Fritzell et al. 2014; Hillemeier et al. 2003; Jachimowicz et al. 2020; Kravdal 2008; Marmot and Wilkinson 2001; Rambotti 2015). Fourth, studies juxtapose the effects of one’s own income against the effects of the mean incomes of a place/community or reference group (Asadullah and Chaudhury 2012; D’Ambrosio, Jantti, and Lepinteur 2020; Daly, Wilson, and Johnson 2013; Luttmer 2005).

While all these types of studies have been valuable, each has limitations. The first type is vulnerable because of the dubious external validity of laboratory experiments. Also, laboratory simulations of income or consumption are unlikely to be as construct valid as measures of actual economic resources. For the second type, it is unclear if subjective perceptions of relative deprivation in observational studies actually track objective material deprivation. While the third type has generated a voluminous literature, many question these ecological studies’ typically macro-level cross-sectional research designs, the overreliance on bivariate correlations, the vulnerability to omitted variable bias, and the non-robustness of results (Beckfield 2004; Curran and Mahutga 2018; Kravdal 2008).

The fourth approach avoids some of these problems. However, even those analyses are unable to measure both absolute and relative income at the person level. The mean income of a place or reference group confounds two very different concepts. On one hand, it captures the prevailing standards of a place or reference group – and a negative coefficient therefore tracks relative deprivation. On the other hand, the mean of a place or group includes a wide variety of unobserved characteristics of places or groups. In particular, the mean of a place or group includes the individual characteristics driving selection into places or groups.

To be concrete about the present study’s contribution, we emphasize two innovations. In the process, we contrast our study with the relatively few similar prior panel analyses.

First we exploit within-person, within-age, and within-time variation in the United States. Heretofore, the literature has relied far more on cross-sectional analysis than on longitudinal analyses. Compared to the many cross-sectional studies, there are few panel analyses comparing the competing effects of absolute and relative income *within* individuals. One advantage of within-individual analyses is that one can net out stable unobserved between-person characteristics, many of which could confound the relationship between income and well-being. A second advantage is that absolute and relative income are highly correlated in cross-sectional data, while there is far greater differentiation over time and when they are differenced from within-person means (Brady et al. 2022). For instance, in the 2019 cross section of our data, the bivariate correlation between our measures of relative and absolute income is 0.90.

Prior longitudinal analyses are certainly valuable. Panel analyses have been conducted on British data (Boyce, Brown, and Moore 2010; Brown, Gray, and Roberts 2015; Daly, Boyce, and Wood 2015; Hounkpatin et al. 2015; Jones and Wildman 2008); German data (Ferrer-i-Carbonell 2005; Ferrer-i-Carbonell and Frijters 2004; Headey, Muffels, and Wagner 2010); and Australian, British, Dutch, German, and Hungarian data (Headey, Muffels, and Wooden 2008). Nevertheless, we have been able to identify only one unpublished working paper with a panel analysis of the United States (Brown, Gathergood, and Weber 2017).^4^ The United States has higher income inequalities, worse and more unequal population health, and a weaker welfare state than most rich democracies. Hence the United States is an important case that deserves study in its own right.

We also use higher-quality state-of-the-art income measures, incorporating taxes and transfers and adjusting for household size. Our measures of “post-fisc” equivalized income follow prevailing international measurement standards (Brady et al. 2018; Brady et al. 2022; Brady and Parolin 2020; Duncan et al. 2002; Rainwater and Smeeding 2004). Transfers and tax credits smooth and stabilize consumption and improve short- and long-term well-being (Brady and Parolin 2020; Hoynes, Schanzenbach, and Almond 2016; Rainwater and Smeeding 2004). Including taxes and tax credits is also particularly crucial in the United States given the central social policy role of the earned income and child tax credits. Post-fisc equivalized income far outperforms cruder measures of income (or wealth, occupation, or earnings) as a proxy for permanent/long-term income (Brady et al. 2018), is far more consequential to subsequent life chances (Brady et al. 2020; Brady et al. 2022), and better explains Black–White inequalities (Brady et al. 2020).

Most prior cross-sectional studies have neglected these prevailing international standards in income measurement. Indeed, many use proxies for relative income, such as relative deprivation (Christoph 2010; Stewart 2006) or employment grades (Marmot et al. 1991), and then compare those measures – but not relative income – against absolute income. Almost all similar panel analyses provide remarkably little information about how income is measured (Boyce, Brown, and Moore 2010; Brown, Gray and Roberts 2015; Ferrer-i-Carbonell 2005; Ferrer-i-Carbonell and Frijters 2004; Headey, Muffels, and Wooden 2008; Hounkpatin et al. 2015; Jones and Wildman 2008). As a result, it is routinely unclear what the income concept is. In the few studies that clearly define income, scholars use gross income, including earnings and transfers, but omit taxes and tax credits (e.g., Brown, Gathergood, and Weber 2017; Daly, Boyce, and Wood 2015). Most relevant studies (Brown, Gathergood, and Weber 2017; Daly, Boyce, and Wood 2015; Ferrer-i-Carbonell 2005; Ferrer-i-Carbonell and Frijters 2004) have no mention of equivalizing for household size, and some use unequivalized income (e.g., Hounkpatin et al. 2015). Very few explicitly equivalize for household size.

## 4. Methods

### 4.1 Data and sample

We use the Panel Study of Income Dynamics (PSID), which is ideal because of its large, nationally representative sample and multiple health outcomes observed at numerous time points (PSID 2019). Furthermore, our use of the Cross-National Equivalent File (CNEF) – a supplement and extension to the PSID – distinctively provides higher-quality income measures (Frick et al. 2007). The PSID requires registration to access the data. However, our replication code will be publicly available on the first author’s website. The code for assembling our dataset from the PSID and the CNEF is also available on the first author’s website (Brady and Kohler 2022).

Our analytic sample includes all observations with valid health and income measures through the 2019 wave. The number of valid cases differs by outcome. Across outcomes, the main analyses include 12,361–17,094 individuals for 60,317–198,396 person-years across waves (annual through 1997; biannual after) from 1984 to 2019.^5^

Our main analyses include all adult respondents. This includes “heads” with self-reports and spouses with proxy reports. We analyze heads only in Table A–3. Most PSID heads are men, and the female heads are less likely to be partnered, married, and high school or college graduates. Obviously, there are also well-established significant sex differences in health. Therefore we report separate models by sex (Table A–5). Further, because health exhibits greater heterogeneity among older respondents (Brady et al. 2022), we also show models among those aged 40-plus (Table A-6). As demonstrated below, the results are robust in these alternative samples.

### 4.2 Measures

The variable coding and descriptive statistics are shown in Table A–1. The dependent variables include four outcomes available across many PSID waves. Unlike most prior studies, which focus on just one outcome, we emulate the smaller set of studies incorporating multiple health and well-being outcomes (e.g., Brown, Gray, and Roberts 2015; Chin 2010; Daly, Boyce, and Wood 2015; Jones and Wildman 2008).

For general health status, two outcomes are derived from the standard five-point self-rated health item (Chin 2010; Daly, Boyce, and Wood 2015; Franks and Gold 2003; Jones and Wildman 2008; Schnittker and Bacak 2014): *self-rated health* (1 = poor, 2 = fair, 3 = good, 4 = very good, 5 = excellent) and *poor self-rated health* (0 = excellent, very good, and good; 1 = fair and poor) (Mackenbach et al. 1997; Manor, Matthews, and Power 2000). We also use Kessler et al.’s K-6 nonspecific scale (Kessler et al. 2002) for *psychological distress* (scored 0–24). *Life satisfaction* is measured using a single item on a five-point scale: “Please think about your life as a whole. How satisfied are you with it?” (1 = not at all satisfied to 5 = completely satisfied) (Boyce, Brown, and Moore 2010; Brown, Gathergood, and Weber 2017; Brown, Gray, and Roberts 2015; Chin 2010; Diener et al. 1993; Ferrer-i-Carbonell 2005; Headey, Muffels, and Wooden 2008; Headey, Muffels, and Wagner 2010).

Our key independent variables – relative and absolute income – use post-fisc equivalized income. These incorporate taxes, tax credits (e.g., the Earned Income Tax Credit), cash transfers (e.g., Temporary Assistance for Needy Families), and near cash transfers (e.g., the Supplemental Nutrition Assistance Program). They adjust for household size by dividing by the square root of household members. Thus our measures explicitly improve on the conventional income measures in this literature, such as gross (pre-fisc) non-equivalized income.

For *absolute income*, we measure post-fisc equivalized income in real inflation-adjusted 2021 dollars. Following prior studies, we convert absolute income to natural log. Table A–4 shows similar results with absolute income not logged. For *relative income*, we measure post-fisc equivalized income in rank percentiles. These absolute and relative measures are similar to those in related studies of health and well-being (Boyce, Brown, and Moore 2010; Brady et al. 2022; Brown, Gathergood, and Weber 2017; Hounkpatin et al. 2015; Ladin, Daniels, and Kawachi 2010; Subramanyam et al. 2009), consistent with relative and absolute poverty measures (Brady and Parolin 2020; Gustafsson and Sai 2020; Rainwater and Smeeding 2004; Smeeding 2016), and often employed in intergenerational analyses (Brady et al. 2020; Fox, Torche, and Waldfogel 2016). To illustrate the differences between absolute and relative income, a person with an (unlogged) equivalized absolute income of $31,000 would vary, being at about the 67^th^ percentile in 1980, the 57^th^ percentile in 1990, the 49^th^ percentile in 2001, and the 50^th^ percentile in 2015.

The analyses also include five-year *age* categories and *wave* (survey year) as fixed effects (FEs). Some models adjust for education and marital status as time-varying covariates (see Table A–1). We intentionally omit additional controls because we aim to strictly avoid post-treatment bias. That is, we intentionally avoid adjusting for any variable that could mediate the relationship between income and outcomes (e.g., health behavior, and wealth). Because the models estimate within-person effects, they also omit time-invariant characteristics like sex (but see Table A–5), race/ethnicity, and stable traits.

### 4.3 Analytical strategy

The main analysis utilizes three-way fixed effects models:

$${\text{Y}}_{\text{ijt}}=\beta_{0}+\;{\beta \text{Relative }}_{\text{ijt}}+{\beta \text{Absolute }}_{\text{ijt}}+{\beta \text{P}}_{\text{i}}+{\beta \text{Z}}_{\text{j}}+{\beta \text{W}}_{\text{t}}+{\beta \text{X}}_{\text{ijt}}+\varepsilon_{\text{ijt}}.$$

Each outcome *Y* varies within individuals *i*, who are nested in five-year age categories *j* and have multiple observations over survey waves *t*. Each outcome is a function of both relative and absolute income. The individual person FEs *Pi* net out individuals’ stable unobserved characteristics; five-year age categories *Zj* net out age differences; and time/wave *Wt* nets out population-wide changes over time. As a result, the models concentrate on variation within person, age, and time. Again, this approach offers two major advantages. First, the FEs remove stable between-person, between-age, and between-year unobserved heterogeneities with stable effects. Second, within-person variation offers much greater differentiation between relative and absolute income.

The use of three-way FEs does require special attention, however. Therefore we estimate all models with and without the wave FEs (βWt). Person FEs are perfectly collinear with cohort FEs, so the classic age-period-cohort problem is triggered when person, age, and wave FEs are all included. We reduce this problem because the five-year age FEs differ from yearly age FEs. In addition, with wave FEs, absolute income is demeaned from each year’s mean absolute income (Absolute_ijt_ – [Mean Absolute_ij_]). This converts absolute income into a within-time measure, which is not strictly consistent with the concept of “absolute” and becomes closer to “relative.” Hence including wave FEs changes the interpretation of absolute income. Fortunately, the results are consistent regardless of whether we include wave FEs.

All models are linear, including the binary outcome poor health, for at least three reasons. First, FE conditional logit models drop individuals who lack variation in a dependent variable. Indeed, approximately 60% of individuals (and about 50% of observations) would be dropped from the poor health models because their outcome does not change. We strongly prefer to retain as many individuals as possible, including those with stable health and well-being. Indeed, if relative income or absolute income changes but health or well-being does not, this is certainly relevant. Second, logit models with FEs are vulnerable to the incidental parameters problem, which could result in bias (see, e.g., Cruz-Gonzalez, Fernandez-Val, and Weidner 2017). Third, the results are more easily interpreted and compared across outcomes and models. We also use robust standard errors clustered at the individual level.

## 5. Results

### 5.1 Main analyses

**Figure 1:**
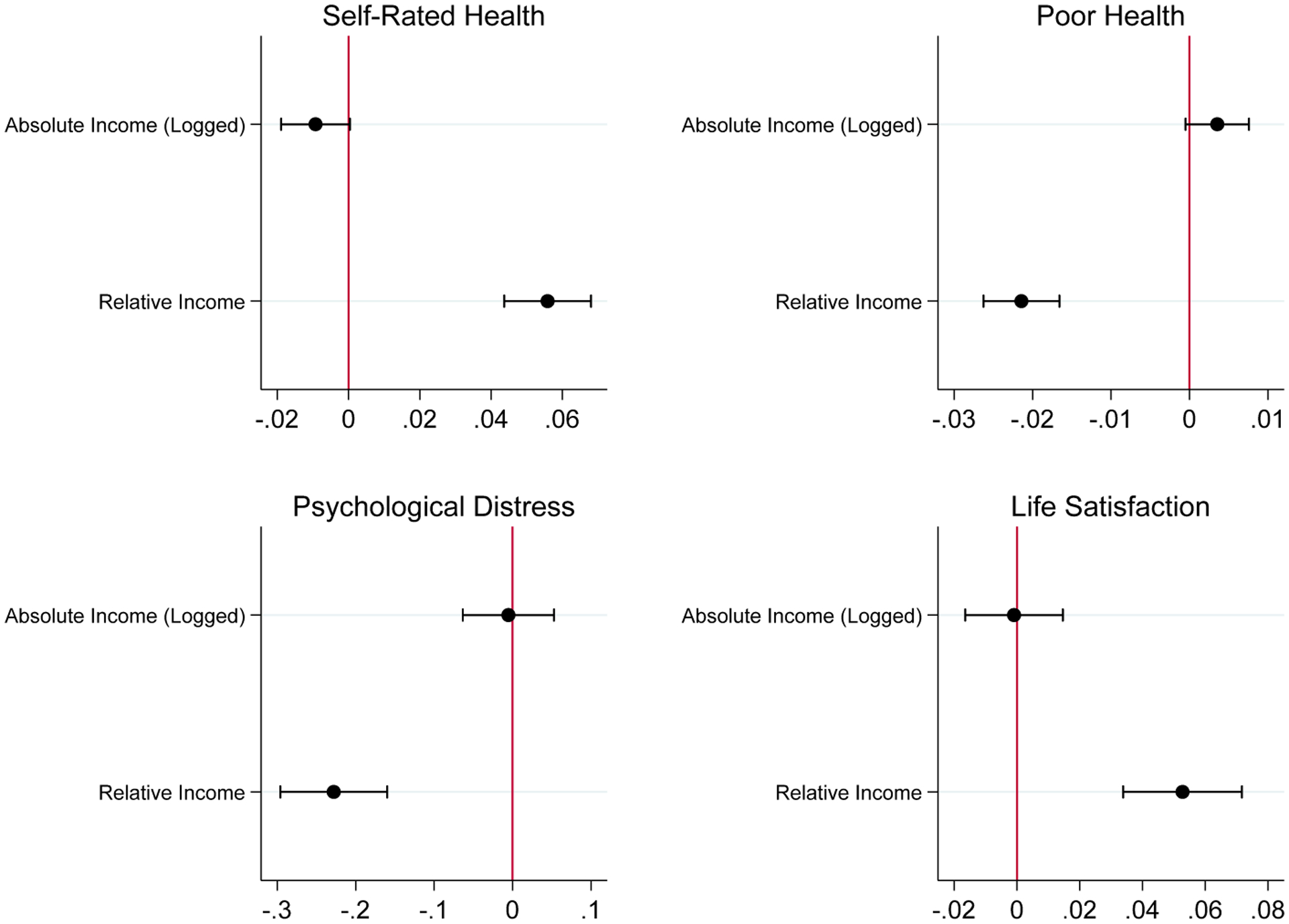
X-standardized coefficients for absolute and relative income for each outcome (see Table A-2, model 4) *Notes*: Dots are point estimates, horizontal lines are 95% CIs, and vertical lines mark zero.

For each outcome, Figure 1 displays the x-standardized coefficients (x-stdBs) and 95% confidence intervals (CIs) for absolute and relative income. These results are from the fourth models in Table A-2. Those tables include alternative specifications, but because the results are consistent, we focus on the fourth models.

Relative income predicts all four outcomes with 99.9% CIs not overlapping zero. By contrast, absolute income’s 95% CIs always overlap zero. The relative income coefficients are always signed toward better health and well-being. However, the absolute income coefficients are signed for worse self-rated health and poor health.^6^ In terms of substantive magnitude, the x-stdBs for relative income are all much larger than for absolute income. For all outcomes, Figure 1 shows that the 95% CIs do not overlap, which is a stringent test of the differences in coefficients.

For self-rated health, a one standard deviation (SD) increase in relative income is associated with 0.06 higher self-rated health and a −0.02 lower probability of poor health. By contrast, a one SD increase in absolute income is associated with only 0.01 lower self-rated health and a 0.004 higher probability of poor health. Hence relative income’s x-stdBs are about 6.2 and 5.3 times larger than those of absolute income for self-rated health and poor health.

For psychological distress, the x-stdB for relative income (−0.23) is far larger (46x) than the x-stdB for absolute income (−0.005). For life satisfaction, the x-stdB for relative income (0.05) is far greater (53x) than the absolute income x-stdB (−0.001).

The largest x-stdBs for relative income are for psychological distress. A one SD increase in relative income is associated with 0.23 SD lower psychological distress. The next largest stdBs are for self-rated health (0.06), life satisfaction (0.05), and poor health (−0.02).

For concrete substantive interpretation, note that a one SD change in relative income corresponds to 28.4 percentage points. Therefore moving from the 25^th^ to the 75^th^ percentile corresponds to roughly a 1.76 SD change in relative income. If relative income moved from the 25^th^ to the 75^th^ percentile, self-rated health would be expected to increase by 0.10 (on mean of 3.5; see Table A-1), the probability of poor health would be expected to decline by 0.04 (on mean of 0.2), psychological distress would be expected to decline by 0.40 (on mean of 3.1), and life satisfaction would be expected to increase by 0.09 (on mean of 3.9). With a few exceptions, across dependent variables, the x-stdBs for relative income are larger than or at least comparable with the coefficients for being a high school or college graduate (versus lacking a high school degree) and being married (versus being single).

### 5.2 Supplementary analyses

Table A–2 displays three alternative specifications of the models: (1) omitting wave/year FEs and controls, (2) omitting wave FEs but including controls, and (3) omitting controls but including wave FEs. All three are generally consistent with Figure 1 (the fourth models). Across models, relative income is much more robust, with 99.9% CIs that never overlap with zero. Also, relative income’s stdBs are always much larger than absolute income’s stdBs. Absolute income’s 95% CIs do not overlap with zero for self-rated health before including wave FEs, but the CIs always overlap with zero for the other three outcomes.

Table A–3 shows models including only self-reports from heads (dropping proxy reports for spouses). All models are similar to Figure 1 in terms of the magnitude of stdBs and CIs.

Table A–4 shows results with absolute income not logged. As in Figure 1, relative income always has 99.9% CIs that do not overlap with zero and much larger stdBs than absolute income for all outcomes. However, absolute income’s 95% CIs do not overlap with zero for self-rated health, and its 99% or 99.9% CIs do not overlap with zero for poor health. Nevertheless, absolute income’s CIs do overlap with zero for psychological distress and life satisfaction, and its stdBs are always far smaller than relative income.

Table A–5 shows largely consistent results when we stratify the fourth models by sex. For both sexes and for all outcomes, relative income has much larger stdBs than absolute income, and relative income’s CIs never overlap with zero.

Table A–6 includes only respondents aged 40-plus to focus on older respondents, who tend to have more heterogeneity in health and well-being. Again, relative income’s stdBs are substantively larger than those of absolute income. Again, relative income’s 99% CIs never overlap with zero. By contrast, absolute income’s 95% CIs do not overlap with zero only for self-rated health and poor health, and both are in the wrong direction (negative for self-rated health and positive for poor health).

Finally, Table A–7 shows the final models, but absolute and relative income are entered in separate models. Throughout, we have advocated for including both in the same model, as this provides a more direct comparison and test. That said, even if we enter the two income variables separately, the results buttress our conclusions. Relative income always has much larger x-stdBs than absolute income. Indeed, for all four outcomes, relative income’s x-stdBs are more than 1.9x as large as absolute income’s x-stdBs. In these separate models, the CIs for absolute income never overlap with zero, and none of the absolute income coefficients are signed in the wrong direction. Thus, without adjusting for relative income, absolute income can be shown to enhance well-being. Still, the most important pattern is that the x-stdBs for relative income are always much larger than the x-stdBs for absolute income.

## 6. Conclusion

A classic unresolved debate concerns whether absolute income or relative income is more consequential for health and well-being. While absolute income is constant across time and place, relative income is contextualized as a hierarchical location within the distribution of time and place. This debate is central to inequalities and deprivation, norms about distributive justice, poverty measurement, and income-based interventions for health. It also should inform how scholars measure income in their analyses.

Most of the literature uses cross-sectional data, and comparatively few studies examine variation within individuals over time. Prior longitudinal analyses have used weaker income measures. To the best of our knowledge, there has been only one unpublished panel analysis within the United States. By contrast, we exploit higher-quality income measures and multiple measures of health and well-being in the PSID-CNEF. Our three-way FE approach scrutinizes within-person/within-age/within-time variation and better differentiates between absolute and relative income.

Our results demonstrate that relative income is more important than absolute income to health and well-being. For all four outcomes, relative income has much larger x-stdBs and far smaller *p*-values than absolute income. Indeed, the comparative magnitude of the x-stdBs overwhelmingly suggests that relative income is more important than absolute income. Our results are also robust to a wide variety of alternative model specifications and samples.

Because our three-way FE approach nets out stable between-individual, between–time period, and between-age differences in health, our study contributes further convincing evidence of long-standing claims that income is a fundamental cause of better health (Deaton 2014; Headey, Muffels, and Wagner 2010; Link and Phelan 1995; Mackenbach et al. 2005; Subramanyam et al. 2009). Uniquely, we provide a useful test demonstrating that it is relative income that is most important to health and well-being. Notably, some influential prior studies have concluded that income has quite small effects on health and well-being while measuring only absolute income and omitting relative income (e.g., Diener et al. 1993; Ferrer-i-Carbonell 2005; Ferrer-i-Carbonell and Frijters 2004). Our evidence suggests that one will underestimate the effects of income on health and well-being if income is measured only absolutely and not relatively (see also Ball and Chernova 2007; Boyce, Brown, and Moore 2010; Brady et al. 2022; Brown, Gathergood, and Weber 2017; Christoph 2010; Hounkpatin et al. 2015; Marmot and Wilkinson 2001). Further, because relative income is closer to inequality and absolute income is closer to economic development, we provide novel evidence for and a productive direction by which to study the consequences of inequality (Avendano 2012; Hastings 2019; Marmot and Wilkinson 2001; Subramanyam et al. 2009). Even though rising economic development benefits population health, the benefits of economic development are constrained by inequality (Curran and Mahutga 2018).

Our study has limitations that future research should address. We suggest five issues. First, the PSID added a greater variety of health outcomes only recently. As more time points become available for outcomes like chronic conditions and acute events, our approach should be replicated. Second, we split the analyses only by sex and age, but others can decompose our analyses further by other characteristics, such as race/ethnicity. Third, we examine absolute and relative income across the entire income distribution. It would be worthwhile to focus on lower-income populations to assess if our conclusions apply when distinguishing between relative and absolute poverty (e.g., Brady, Kohler, and Zheng 2023). Fourth, obviously income is not the only salient measure of economic resources. There is evidence that, for example, wealth and consumption are quite consequential as well (e.g., D’Ambrosio, Jantti, and Lepinteur 2020; Headey, Muffels, and Wooden 2008; but see Brady et al. 2020). Our empirical strategy should be applied to other measures to further adjudicate between relative versus absolute economic resources.

Fifth, we have advocated for our three-way FE approach. We have explained that within-individual analyses can net out stable unobserved between-person characteristics, many of which could confound the relationship between income and well-being. Also, because absolute and relative income are highly correlated in cross-sectional data, our approach enables clearer differentiation between the two. Despite what we view as clear merits, we acknowledge reasonable concern about whether within-person analyses can always generalize to between-person comparisons. Therefore there is a need for further research contrasting the well-being of people who vary in terms of absolute and relative income. The perennial challenges in clearly differentiating between absolute and relative income and unobserved confounding remain. Still, to augment the generalization that relative income better predicts well-being, between-person analyses are necessary.

Despite these limitations and needs for further research, we propose that relative income should be centered over absolute income in health and well-being research and maybe even inequality research generally. Fortunately, it is feasible to calculate relative income in almost any dataset, as all it requires is to locate a case within the sample’s distribution. If cross-sectional data are used or it is not possible to include both relative and absolute income in the models, relative income should be prioritized. Relative income appears to be the more salient dimension of inequality and better captures the returns to affluence that higher incomes provide. Economic resources are positional goods, and income’s value for health and well-being is relational to and conditional on others’ resources.

## Footnotes

4 Personal correspondence with Brown confirms that it remains a working paper. That working paper uses the Panel Study of Income Dynamics, as we do, but it does not use the higher-quality income measures from the Cross-National Equivalent File. Another study analyzes the Wisconsin Longitudinal Study (Hounkpatin et al. 2015), which is a panel analysis but not nationally representative of the United States. Further, the study does not use panel models. One reported model simply analyzes the 1992–1993 cross section. Another regresses the 2003–2005 outcomes on the 1992–1993 independent variables. As a result, and because absolute and relative income are highly correlated in a cross section, it does not include both measures in one model and instead mainly compares fit statistics across separate models.

5 In analyses available upon request, we tested the sensitivity of our results in the broadly defined tails of the income distribution (the top and bottom 10%, 25%, and 33%). We found that, compared to the entirety of the distribution, absolute income and relative income in the tails were fairly similarly highly correlated. We also found broadly similar patterns in the regression models.

6 Although we report coefficients for absolute income that are signed in the wrong direction, we caution against emphasizing these results. The main pattern across the main results and supplementary analyses is that absolute income does not worsen health and well-being. The abstract precisely states that absolute income coefficients are occasionally signed in the wrong direction. Mostly, absolute income’s coefficients have confidence intervals that overlap with zero, and they are substantively smaller coefficients than those of relative income.

## Appendix

**Table A-1:** Descriptive statistics and descriptions of variables for main analyses of PSID and CNEF, 1984–2019

	Mean	SD	N	Description
***Dependent variables***				
Self-rated health	3.52	1.08	201,920	1 = poor, 2 = fair, 3 = good, 4 = very good, 5 = excellent
Poor health	0.17	0.37	201,920	0 = excellent, very good, and good; 1 = fair and poor
Psychological distress	3.08	3.81	66,874	K-6 non-specific scale, scored 0–24
Life satisfaction	3.85	.82	52,258	1 = not at all satisfied, 2 = not very satisfied, 3 = somewhat satisfied, 4 = very satisfied, 5 = completely satisfied
***Independent variables***				
Absolute income (logged)	10.49	1.10	201,920	Post-fisc equivalized income in real inflationadjusted 2021 dollars
Relative income	56.81	28.36	200,772	Post-fisc equivalized income in yearly rank percentiles
Education				
* High School Grad*.	0.84	0.37	200,772	0–1
* College Grad*.	0.26	0.44	201,920	0–1
Married/partnered (vs. single)	0.64	0.48	140,252	0–1
Age	48.74	16.98	201,920	5-year FEs in analyses

**Table A-2:** Main regression results a) Self-rated health: Regression coefficients and robust standard errors in parentheses, PSID-CNEF (1984–2019)

	(1)	(2)	(3)	(4)
Absolute income (logged)	−0.010	−0.010	−0.009	−0.009
	(0.005)	(0.005)	(0.005)	(0.005)
Relative income	0.051	0.054	0.053	0.056
	(0.006)	(0.006)	(0.006)	(0.006)
H.S. Graduate^a^		−0.051		−0.029
		(0.025)		(0.025)
College Graduate^a^		−0.006		0.014
		(0.022)		(0.023)
Married/partnered^b^		−0.043		−0.044
		(0.011)		(0.011)
N	198,396	198,396	198,396	198,396
Individuals	17,094	17,094	17,094	17,094
Age FEs	YES	YES	YES	YES
Wave FEs	NO	NO	YES	YES

*Note*: X-standardized coefficients for relative and absolute income.

a Referent = less than high school.

b Referent = single.

**Table A-2:** Main regression results b) Poor health: Regression coefficients and robust standard errors in parentheses, PSID-CNEF (1984–2019)

	(1)	(2)	(3)	(4)
Absolute income (logged)	0.004	0.004	0.004	0.004
	(0.002)	(0.002)	(0.002)	(0.002)
Relative income	−0.021	−0.021	−0.021	−0.021
	(0.002)	(0.002)	(0.002)	(0.002)
H.S. Graduate^a^		0.032		0.028
		(0.010)		(0.010)
College graduate^a^		−0.009		−0.013
		(0.009)		(0.009)
Married/partnered^b^		0.000		0.000
		(0.004)		(0.004)
N	198,396	198,396	198,396	198,396
Individuals	17,094	17,094	17,094	17,094
Age FEs	YES	YES	YES	YES
Wave FEs	NO	NO	YES	YES

*Note*: X-standardized coefficients for relative and absolute income.

a Referent = less than high school.

b Referent = single.

**Table A-2:** Main regression results c) Psychological distress: Regression coefficients and robust standard errors in parentheses, PSID-CNEF (1984–2019)

	(1)	(2)	(3)	(4)
Absolute income (logged)	−0.002	−0.001	−0.006	−0.005
	(0.030)	(0.030)	(0.030)	(0.030)
Relative income	−0.239	−0.233	−0.234	−0.228
	(0.035)	(0.035)	(0.035)	(0.035)
H.S. graduate^a^		0.074		0.037
		(0.102)		(0.103)
College graduate^a^		0.074		0.060
		(0.078)		(0.079)
Married/partnered^b^		−0.198		−0.197
		(0.065)		(0.065)
N	77,436	77,436	77,436	77,436
Individuals	13,110	13,110	13,110	13,110
Age FEs	YES	YES	YES	YES
Wave FEs	NO	NO	YES	YES

*Note*: X-standardized coefficients for relative and absolute income.

a Referent = less than high school.

b Referent = single.

**Table A-2:** Main regression results d) Life satisfaction: Regression coefficients and robust standard errors in parentheses, PSID-CNEF (1984–2019)

	(1)	(2)	(3)	(4)
Absolute income (logged)	−0.004	−0.004	−0.001	−0.001
	(0.008)	(0.008)	(0.008)	(0.008)
Relative income	0.062	0.056	0.059	0.053
	(0.010)	(0.010)	(0.010)	(0.010)
H.S. graduate^a^		0.068		0.050
		(0.030)		(0.030)
College graduate^a^		0.001		−0.020
		(0.023)		(0.023)
Married/partnered^b^		0.236		0.236
		(0.020)		(0.020)
N	60,317	60,317	60,317	60,317
Individuals	12,361	12,361	12,361	12,361
Age FEs	YES	YES	YES	YES
Wave FEs	NO	NO	YES	YES

*Note*: X-standardized coefficients for relative and absolute income.

a Referent = less than high school.

b Referent = single.

**Table A-3:** Regression results for self-reported heads only a) Self-rated health: Regression coefficients and robust standard errors in parentheses, head reporting only, PSID-CNEF (1984–2019)

	(1)	(2)	(3)	(4)
Absolute income (logged)	−0.009	−0.010	−0.010	−0.010
	(0.006)	(0.006)	(0.006)	(0.006)
Relative income	0.056	0.057	0.058	0.058
	(0.008)	(0.008)	(0.008)	(0.008)
H.S. graduate^a^		−0.053		−0.031
		(0.030)		(0.031)
College graduate^a^		−0.018		0.002
		(0.029)		(0.029)
Married/partnered^b^		−0.003		−0.002
		(0.016)		(0.016)
N	136,935	136,935	136,935	136,935
Individuals	13,887	13,887	13,887	13,887
Age FEs	YES	YES	YES	YES
Wave FEs	NO	NO	YES	YES

*Note*: X-standardized coefficients for relative and absolute income.

a Referent = less than high school.

b Referent = single.

**Table A-3:** Regression results for self-reported heads only b) Poor health: Standardized regression coefficients and robust standard errors in parentheses, head reporting only, PSID-CNEF (1984–2019)

	(1)	(2)	(3)	(4)
Absolute income (logged)	0.004	0.005	0.005	0.005
	(0.002)	(0.002)	(0.002)	(0.002)
Relative income	−0.023	−0.024	−0.024	−0.024
	(0.003)	(0.003)	(0.003)	(0.003)
H.S. graduate^a^		0.028		0.024
		(0.012)		(0.012)
College graduate^a^		0.008		0.003
		(0.012)		(0.012)
Married/partnered^b^		−0.008		−0.008
		(0.005)		(0.005)
N	136,935	136,935	136,935	136,935
Individuals	13,887	13,887	13,887	13,887
Age FEs	YES	YES	YES	YES
Wave FEs	NO	NO	YES	YES

*Note*: X-standardized coefficients for relative and absolute income.

a Referent = less than high school.

b Referent = single.

**Table A-3:** Regression results for self-reported heads only c) Psychological distress: Regression coefficients and robust standard errors in parentheses, head reporting only, PSID-CNEF (1984–2019)

	(1)	(2)	(3)	(4)
Absolute income (logged)	−0.033	−0.033	−0.035	−0.035
	(0.033)	(0.033)	(0.033)	(0.033)
Relative income	−0.127	−0.125	−0.124	−0.121
	(0.043)	(0.043)	(0.043)	(0.043)
H.S. graduate^a^		−0.032		−0.077
		(0.158)		(0.160)
College graduate^a^		−0.034		−0.050
		(0.107)		(0.109)
Married/partnered^b^		−0.098		−0.095
		(0.097)		(0.097)
N	45,226	45,226	45,226	45,226
Individuals	8,372	8,372	8,372	8,372
Age FEs	YES	YES	YES	YES
Wave FEs	NO	NO	YES	YES

*Note*: X-standardized coefficients for relative and absolute income.

a Referent = less than high school.

b Referent = single.

**Table A-3:** Regression results for self-reported heads only d) Life satisfaction: Regression coefficients and robust standard errors in parentheses, head reporting only, PSID-CNEF (1984–2019).

	(1)	(2)	(3)	(4)
Absolute income (logged)	−0.002	−0.002	−0.001	−0.001
	(0.009)	(0.009)	(0.009)	(0.009)
Relative income	0.039	0.036	0.037	0.034
	(0.012)	(0.012)	(0.012)	(0.012)
H.S. graduate^a^		0.097		0.068
		(0.062)		(0.062)
College graduate^a^		−0.032		−0.058
		(0.034)		(0.034)
Married/partnered^b^		0.227		0.225
		(0.032)		(0.032)
N	35,637	35,637	35,637	35,637
Individuals	7,781	7,781	7,781	7,781
Age FEs	YES	YES	YES	YES
Wave FEs	NO	NO	YES	YES

*Note*: X-standardized coefficients for relative and absolute income.

a Referent = less than high school.

b Referent = single.

**Table A-4:** Regression models with non-logged absolute income a) Self-rated health: Regression coefficients and robust standard errors in parentheses, PSID-CNEF (1984–2019)

	(1)	(2)	(3)	(4)
Absolute income	0.007	0.007	0.007	0.007
	(0.003)	(0.003)	(0.003)	(0.003)
Relative income	0.039	0.042	0.041	0.044
	(0.005)	(0.005)	(0.005)	(0.005)
H.S. graduate^a^		−0.050		−0.028
		(0.025)		(0.025)
College graduate^a^		−0.005		0.014
		(0.022)		(0.023)
Married/partnered^b^		−0.043		−0.044
		(0.011)		(0.011)
N	198,396	198,396	198,396	198,396
Individuals	17,094	17,094	17,094	17,094
Age FEs	YES	YES	YES	YES
Wave FEs	NO	NO	YES	YES

*Note*: X-standardized coefficients for relative and absolute income.

a Referent = less than high school.

b Referent = single.

**Table A-4:** Regression models with non-logged absolute income b) Poor health: Regression coefficients and robust standard errors in parentheses, PSID-CNEF (1984–2019)

	(1)	(2)	(3)	(4)
Absolute income	−0.003	−0.003	−0.003	−0.003
	(0.001)	(0.001)	(0.001)	(0.001)
Relative income	−0.016	−0.016	−0.017	−0.017
	(0.002)	(0.002)	(0.002)	(0.002)
H.S. graduate^a^		0.032		0.028
		(0.010)		(0.010)
College graduate^a^		−0.009		−0.013
		(0.009)		(0.009)
Married/partnered^b^		0.000		0.001
		(0.004)		(0.004)
N	198,396	198,396	198,396	198,396
Individuals	17,094	17,094	17,094	17,094
Age FEs	YES	YES	YES	YES
Wave FEs	NO	NO	YES	YES

*Note*: X-standardized coefficients for relative and absolute income.

a Referent = less than high school.

b Referent = single.

**Table A-4:** Regression models with non-logged absolute income c) Psychological distress: Regression coefficients and robust standard errors in parentheses, PSID-CNEF (1984–2019)

	(1)	(2)	(3)	(4)
Absolute income	0.013	0.014	0.012	0.012
	(0.025)	(0.025)	(0.025)	(0.025)
Relative income	−0.249	−0.242	−0.247	−0.240
	(0.031)	(0.031)	(0.031)	(0.031)
H.S. graduate^a^		0.074		0.038
		(0.102)		(0.103)
College graduate^a^		0.075		0.060
		(0.078)		(0.079)
Married/partnered^b^		−0.198		−0.197
		(0.065)		(0.065)
N	77,436	77,436	77,436	77,436
Individuals	13,110	13,110	13,110	13,110
Age FEs	YES	YES	YES	YES
Wave FEs	NO	NO	YES	YES

*Note*: X-standardized coefficients for relative and absolute income.

a Referent = less than high school.

b Referent = single.

**Table A-4:** Regression models with non-logged absolute income d) Life satisfaction: Regression coefficients and robust standard errors in parentheses, PSID-CNEF (1984–2019)

	(1)	(2)	(3)	(4)
Absolute income	−0.005	−0.006	−0.004	−0.004
	(0.005)	(0.005)	(0.005)	(0.005)
Relative income	0.062	0.056	0.061	0.055
	(0.008)	(0.008)	(0.008)	(0.008)
H.S. graduate^a^		0.068		0.050
		(0.030)		(0.030)
College graduate^a^		0.001		−0.020
		(0.023)		(0.023)
Married/partnered^b^		0.236		0.236
		(0.020)		(0.020)
N	60,317	60,317	60,317	60,317
Individuals	12,361	12,361	12,361	12,361
Age FEs	YES	YES	YES	YES
Wave FEs	NO	NO	YES	YES

*Note*: X-standardized coefficients for relative and absolute income.

a Referent = less than high school.

b Referent = single.

**Table A-5:** Final regression models split by sex a) Self-rated health: Regression coefficients and robust standard errors in parentheses, PSID-CNEF (1984–2019)

	Men	Women
Absolute income (logged)	−0.003	−0.015
	(0.007)	(0.007)
Relative income	0.052	0.061
	(0.009)	(0.009)
H.S. graduate^a^	−0.056	−0.016
	(0.034)	(0.034)
College graduate^a^	0.010	0.011
	(0.036)	(0.029)
Married/partnered^b^	−0.006	−0.070
	(0.016)	(0.015)
N	85,475	112,919
Individuals	7,689	9,406
Age FEs	YES	YES
Wave FEs	YES	YES

*Note*: X-standardized coefficients for relative and absolute income.

a Referent = less than high school.

b Referent = single.

**Table A-5:** Final regression models split by sex b) Poor health: Regression coefficients and robust standard errors in parentheses, PSID-CNEF (1984–2019)

	Men	Women
Absolute income (logged)	−0.003	−0.015
	(0.002)	(0.003)
Relative income	−0.023	−0.021
	(0.003)	(0.004)
H.S. graduate^a^	0.044	0.017
	(0.014)	(0.014)
College graduate^a^	−0.010	−0.015
	(0.013)	(0.012)
Married/partnered^b^	−0.006	0.006
	(0.006)	(0.006)
N	85,475	112,919
Individuals	7,689	9,406
Age FEs	YES	YES
Wave FEs	YES	YES

*Note*: X-standardized coefficients for relative and absolute income.

a Referent = less than high school.

b Referent = single.

**Table A-5:** Final regression models split by sex c) Psychological distress: Regression coefficients and robust standard errors in parentheses, PSID-CNEF (1984–2019)

	Men	Women
Absolute income (logged)	−0.008	−0.008
	(0.041)	(0.043)
Relative income	−0.172	−0.273
	(0.050)	(0.048)
H.S. graduate^a^	−0.104	0.178
	(0.141)	(0.150)
College graduate^a^	0.105	0.040
	(0.117)	(0.108)
Married/partnered^b^	−0.087	−0.292
	(0.096)	(0.087)
N	35,080	42,352
Individuals	6,111	7,000
Age FEs	YES	YES
Wave FEs	YES	YES

*Note*: X-standardized coefficients for relative and absolute income.

a Referent = less than high school.

b Referent = single.

**Table A-5:** Final regression models split by sex d) Life satisfaction: Regression coefficients and robust standard errors in parentheses, PSID-CNEF (1984–2019)

	Men	Women
Absolute income (logged)	0.000	−0.003
	(0.010)	(0.012)
Relative income	0.041	0.064
	(0.014)	(0.013)
H.S. graduate^a^	0.054	0.048
	(0.039)	(0.046)
College graduate^a^	−0.080	0.025
	(0.036)	(0.030)
Married/partnered^b^	0.227	0.243
	(0.031)	(0.027)
N	27,253	33,058
Individuals	5,723	6,637
Age FEs	YES	YES
Wave FEs	YES	YES

*Note*: X-standardized coefficients for relative and absolute income.

a Referent = less than high school.

b Referent = single.

**Table A-6:** Regression models for respondents 40+ a) Self-rated health: Regression coefficients and robust standard errors in parentheses, older sample (> age 39), PSID-CNEF (1984–2019)

	(1)	(2)	(3)	(4)
Absolute income (logged)	−0.018	−0.018	−0.016	−0.016
	(0.007)	(0.007)	(0.007)	(0.007)
Relative income	0.062	0.064	0.058	0.061
	(0.008)	(0.008)	(0.008)	(0.008)
H.S. graduate^a^		−0.050		−0.042
		(0.041)		(0.041)
College graduate^a^		0.036		0.038
		(0.040)		(0.040)
Married/partnered^b^		−0.057		−0.065
		(0.018)		(0.018)
N	112,864	112,864	112,864	112,864
Individuals	10,512	10,512	10,512	10,512
Age FEs	YES	YES	YES	YES
Wave FEs	NO	NO	YES	YES

*Note*: X-standardized coefficients for relative and absolute income.

a Referent = less than high school.

b Referent = single.

**Table A-6:** Regression models for respondents 40+ b) Poor health: Regression coefficients and robust standard errors in parentheses, older sample (> age 39), PSID-CNEF (1984–2019)

	(1)	(2)	(3)	(4)
Absolute income (logged)	0.008	0.008	0.007	0.007
	(0.003)	(0.003)	(0.003)	(0.003)
Relative income	−0.025	−0.025	−0.024	−0.024
	(0.004)	(0.004)	(0.004)	(0.004)
H.S. graduate^a^		0.029		0.027
		(0.017)		(0.018)
College graduate^a^		−0.012		−0.012
		(0.016)		(0.016)
Married/partnered^b^		0.008		0.010
		(0.007)		(0.007)
N	112,864	112,864	112,864	112,864
Individuals	10,512	10,512	10,512	10,512
Age FEs	YES	YES	YES	YES
Wave FEs	NO	NO	YES	YES

*Note*: X-standardized coefficients for relative and absolute income.

a Referent = less than high school.

b Referent = single.

**Table A-6:** Regression models for respondents 40+ c) Psychological distress: Regression coefficients and robust standard errors in parentheses, older sample (> age 39), PSID-CNEF (1984–2019)

	(1)	(2)	(3)	(4)
Absolute income (logged)	0.002	0.002	0.000	0.000
	(0.036)	(0.036)	(0.036)	(0.036)
Relative income	−0.133	−0.129	−0.134	−0.131
	(0.044)	(0.044)	(0.044)	(0.044)
H.S. graduate^a^		−0.175		−0.205
		(0.209)		(0.210)
College graduate^a^		−0.075		−0.076
		(0.153)		(0.155)
Married/partnered^b^		−0.137		−0.138
		(0.105)		(0.105)
N	38,604	38,604	38,604	38,604
Individuals	6,695	6,695	6,695	6,695
Age FEs	YES	YES	YES	YES
Wave FEs	NO	NO	YES	YES

*Note*: X-standardized coefficients for relative and absolute income.

a Referent = less than high school.

b Referent = single.

**Table A-6:** Regression models for respondents 40+ d) Life satisfaction: Regression coefficients and robust standard errors in parentheses, older sample (> age 39), PSID-CNEF (1984–2019)

	(1)	(2)	(3)	(4)
Absolute income (logged)	0.002	0.002	0.004	0.004
	(0.011)	(0.011)	(0.011)	(0.011)
Relative income	0.044	0.040	0.043	0.039
	(0.013)	(0.013)	(0.014)	(0.014)
H.S. graduate^a^		0.179		0.152
		(0.067)		(0.067)
College graduate^a^		−0.047		−0.069
		(0.044)		(0.044)
Married/partnered^b^		0.264		0.269
		(0.038)		(0.038)
N	29,565	29,565	29,565	29,565
Individuals	6,200	6,200	6,200	6,200
Age FEs	YES	YES	YES	YES
Wave FEs	NO	NO	YES	YES

*Note*: X-standardized coefficients for relative and absolute income.

a Referent = less than high school.

b Referent = single.

**Table A-7:** Separate final regression models for relative and absolute income a) Self-rated health: Regression coefficients and robust standard errors in parentheses with absolute and relative income in separate models, PSID-CNEF (1984–2019)

	(1)	(2)
Absolute income (logged)	0.019	
	(0.004)	
Relative income		0.049
		(0.005)
H.S. graduate^a^	−0.047	−0.029
	(0.025)	(0.025)
College graduate^a^	0.002	0.014
	(0.022)	(0.023)
Married/partnered^b^	−0.033	−0.044
	(0.011)	(0.011)
N	198,396	198,396
Individuals	17,094	17,094
Age FEs	YES	YES
Wave FEs	YES	YES

*Note*: X-standardized coefficients for relative and absolute income.

a Referent = less than high school.

b Referent = single.

**Table A-7:** Separate final regression models for relative and absolute income b) Poor health: Regression coefficients and robust standard errors in parentheses with absolute and relative income in separate models, PSID-CNEF (1984–2019)

	(1)	(2)
Absolute income (logged)	−0.007	
	(0.002)	
Relative income		−0.019
		(0.002)
H.S. graduate^a^	0.031	0.028
	(0.010)	(0.010)
College graduate^a^	−0.012	−0.013
	(0.009)	(0.009)
Married/partnered^b^	−0.004	0.001
	(0.004)	(0.004)
N	198,396	198,396
Individuals	17,094	17,094
Age FEs	YES	YES
Wave FEs	YES	YES

*Note*: X-standardized coefficients for relative and absolute income.

a Referent = less than high school.

b Referent = single.

**Table A-7:** Separate final regression models for relative and absolute income c) Psychological distress: Regression coefficients and robust standard errors in parentheses with absolute and relative income in separate models, PSID-CNEF (1984–2019)

	(1)	(2)
Absolute income (logged)	−0.124	
	(0.023)	
Relative income		−0.232
		(0.026)
H.S. graduate^a^	0.070	0.038
	(0.102)	(0.103)
College graduate^a^	0.065	0.060
	(0.078)	(0.079)
Married/partnered^b^	−0.224	−0.197
	(0.064)	(0.065)
N	77,436	77,436
Individuals	13,110	13,110
Age FEs	YES	YES
Wave FEs	YES	YES

*Note*: X-standardized coefficients for relative and absolute income.

a Referent = less than high school.

b Referent = single.

**Table A-7:** Separate final regression models for relative and absolute income d) Life satisfaction: Regression coefficients and robust standard errors in parentheses with absolute and relative income in separate models, PSID-CNEF (1984–2019)

	(1)	(2)
Absolute income (logged)	0.025	
	(0.006)	
Relative income		0.052
		(0.007)
H.S. graduate^a^	0.065	0.050
	(0.030)	(0.030)
College graduate^a^	0.002	−0.020
	(0.023)	(0.023)
Married/partnered^b^	0.241	0.236
	(0.020)	(0.020)
N	60,317	60,317
Individuals	12,361	12,361
Age FEs	YES	YES
Wave FEs	YES	YES

*Note*: X-standardized coefficients for relative and absolute income.

a Referent = less than high school.

b Referent = single.
